# The Effect of Diel Temperature and Light Cycles on the Growth of *Nannochloropsis oculata* in a Photobioreactor Matrix

**DOI:** 10.1371/journal.pone.0086047

**Published:** 2014-01-20

**Authors:** Bojan Tamburic, Supriya Guruprasad, Dale T. Radford, Milán Szabó, Ross McC Lilley, Anthony W. D. Larkum, Jim B. Franklin, David M. Kramer, Susan I. Blackburn, John A. Raven, Martin Schliep, Peter J. Ralph

**Affiliations:** 1 Plant Functional Biology and Climate Change Cluster, University of Technology Sydney, Sydney, New South Wales, Australia; 2 Department of Biochemistry and Molecular Biology, Michigan State University, East Lansing, Michigan, United States of America; 3 Marine and Atmospheric Research, Commonwealth Scientific and Industrial Research Organisation, Hobart, Tasmania, Australia; 4 Division of Plant Sciences, University of Dundee at the James Hutton Institute, Invergowrie, Scotland, United Kingdom; University of Texas Southwestern Medical Center, United States of America

## Abstract

A matrix of photobioreactors integrated with metabolic sensors was used to examine the combined impact of light and temperature variations on the growth and physiology of the biofuel candidate microalgal species *Nannochloropsis oculata*. The experiments were performed with algal cultures maintained at a constant 20°C versus a 15°C to 25°C diel temperature cycle, where light intensity also followed a diel cycle with a maximum irradiance of 1920 µmol photons m^−2^ s^−1^. No differences in algal growth (Chlorophyll *a*) were found between the two environmental regimes; however, the metabolic processes responded differently throughout the day to the change in environmental conditions. The variable temperature treatment resulted in greater damage to photosystem II due to the combined effect of strong light and high temperature. Cellular functions responded differently to conditions before midday as opposed to the afternoon, leading to strong hysteresis in dissolved oxygen concentration, quantum yield of photosystem II and net photosynthesis. Overnight metabolism performed differently, probably as a result of the temperature impact on respiration. Our photobioreactor matrix has produced novel insights into the physiological response of *Nannochloropsis oculata* to simulated environmental conditions. This information can be used to predict the effectiveness of deploying *Nannochloropsis oculata* in similar field conditions for commercial biofuel production.

## Introduction

### Algal Biofuel Production with *Nannochloropsis*

Microalgae have the potential to produce sustainable and affordable transport fuels for the future. These unicellular photosynthetic organisms capture light energy and use it to fix atmospheric carbon dioxide into proteins, carbohydrates and lipids. The use of algal biofuels leads to considerably lower carbon dioxide emissions per unit energy than for petrochemical-based fuel [1], making algal biofuels a ‘green’ solution to the global energy challenge. Lipid production is the primary focus of the algal biofuels industry [2], [3], but other biofuel precursors such as terpenes are also under development [4]. These secondary metabolites store chemical energy, and they can be chemically processed into biodiesel and aviation fuel [5], [6].

Microalgae of the genus *Nannochloropsis* from the class Eustigmatophyceae are leading candidates for algal biofuel production. The unusual photosynthetic apparatus of these algae contains only chlorophyll *a*. The genome sequence of *Nannochloropsis* species is known, and a genetic transformation method utilising homologous recombination in *Nannochloropsis* has been demonstrated [7], opening up new possibilities in biofuels research using *Nannochloropsis*.

*Nannochloropsis* sp. normally accumulates lipids to 28.7% of the dry weight, or 65 to 70% of the dry weight under nutrient deprivation, resulting in lipid productivities ranging from 25.8 to 60.9 mg L^−1^ d^−1^ [8]–[10]. This productivity compares favourably with other microalgal species that have faster growth rates but lower lipid content, such as *Spirulina maxima* (8.6 mg lipid L^−1^ d^−1^), *Chlorella vulgaris* (9.2 mg lipid L^−1^ d^−1^) and *Dunaliella tertiolecta* (20.0 mg lipid L^−1^ d^−1^) [11]. In general, oil yields from microalgae are several orders of magnitude better than those of higher plants, including traditional biofuel crops such as corn, rape, palm and sugarcane [12].

Many algae operate an active carbon-concentrating mechanism when growing in suboptimal carbon dioxide concentrations. This mechanism transports inorganic carbon from outside the cell to the chloroplast stroma, thereby increasing the rate of photosynthetic carbon fixation. Central to the carbon-concentrating mechanism in *Nannochloropsis* is the uptake of bicarbonate (HCO_3_^−^) across the cell membrane, which results in the accumulation of high intracellular carbon dioxide concentrations, and little benefit was achieved in adding carbon dioxide when attempting to optimise the growth of *N. gaditana* [13].

The alga *Nannochloropsis oculata* was selected for this study based on its ability to grow in ponds of saline, brackish and hypersaline water [8]. This ensures that *N. oculata* biofuel production facilities will never compete with food crops for freshwater or arable land. Australia is perfectly suited for the development of algal biofuels based on *N. oculata* due to its extensive coastline with adjacent marginal or non-arable land, low population density and high sunlight availability [14]. In order to quantify the biofuel production potential of *N. oculata*, it is imperative to understand the physiological processes that govern its growth under naturally variable environmental conditions.

### Photobioreactor (PBR) Matrix

Algae have been grown commercially in outdoor ponds and photobioreactors (PBRs) [15], [16]. Conducting field trials in these large-scale facilities is expensive and time-consuming. A more practical approach is to use laboratory-scale PBRs to simulate natural environmental conditions at the anticipated commercial location; however, most indoor laboratory-scale devices do not fully simulate the outdoor daily cycles of light intensity and temperature [17]–[19]. A series of custom-built PBRs (ePBR, Phenometrics, Lansing, MI, USA) have been integrated with metabolic sensors to simulate outdoor algal growth conditions in the laboratory. Using this innovative technology, it is now possible to mimic diel irradiance and temperature cycles, as well as seasonal variations. The PBR capabilities have been further enhanced with the latest *in vivo* measurement technologies, including dissolved oxygen optodes (bare-fibre oxygen minisensor, Firesting O_2_, PyroScience, Aachen, Germany) and fluorometers (Pocket PAM, Walz, Effeltrich, Germany). It is important to analyse the combined effect of several interacting environmental factors in order to understand how they affect algal growth [20]; therefore, a number of PBRs are operated as a matrix, so that different growth conditions can be explored simultaneously using the same parent culture.

### Light and Temperature Variations

Incident irradiance is the most important environmental factor governing algal biomass production rates [21], [22]. *Nannochloropsis* has been found to have a flexible photosynthetic apparatus, which can acclimate to a wide range of constant and sinusoidally varying light intensities, as well as higher frequency fluctuations in irradiance [23]. The rate of photosynthesis increases linearly with increasing light intensity, and then asymptotically until the photosynthetic electron transport chain, or the enzyme ribulose bisphosphate carboxylase-oxygenase (RuBisCO), becomes light saturated [24], [25]. Irradiances higher than the light saturation level can damage algal cells through photoinhibition [26]. As a result, algal biomass production rates under diel light cycles in outdoor ponds are typically lower than those maintained under constant illumination in the laboratory [27]. On the other hand, *Nannochloropsis* can utilise very intense light provided that subsequent dark periods occur to allow for re-oxidation of the key electron transport components of the photosynthetic apparatus [23]. This light flickering effect can be introduced by controlling the mixing rate in systems grown under natural illumination [23].

Temperature is another important environmental factor because it affects the rate of all enzymatic, electron transport and solute movement reactions within algal cells, and influences the properties of cellular components such as lipids, proteins and carbohydrates [28], [29]. Photosynthesis is one of the most thermally sensitive processes in plants and algae, and it incurs greater damage from occasional extreme temperature events [28], [30]. Aquatic microalgae are frequently forced to adapt to large variations in temperature owing to diel and seasonal cycles, although the temperature differences are not as large as those experienced by desert crust algae or even leaves on vascular plants. These thermal adaptations can determine the variability in biomass and lipid productivity [31]. The optimum temperature for *Nannochloropsis* sp. has been reported as 24–26°C [32], [33]. The effect of temperature variations on the growth rate and photosynthetic performance of *Nannochloropsis* sp. have also been reported [34], [35]. A comprehensive comparison between *N. oculata* growth at constant temperature in the laboratory and at variable temperatures in nature has not been performed. There also remains a distinct knowledge gap in understanding the combined effect of diel light and temperature variations on algal physiology and growth.

### Aims and Objectives

The aim of this study is to compare the growth of *N. oculata* in PBRs (i) at constant temperature (ii) with variable temperature following a diel pattern, where both treatments were held under varying light intensity that also follows the diel cycle. The objective is to understand the combined effects of light and temperature on algal physiology, and to determine the importance of temperature in controlling large-scale biofuel production. Chlorophyll *a* content is measured to determine algal growth rates, dissolved oxygen concentrations and pH are monitored continuously *in situ*, and variable chlorophyll fluorescence and net photosynthesis measurements are used to understand algal physiology.

## Materials and Methods

### Microalgal Culture and Medium

*Nannochloropsis oculata* (Droop) Green (CS-179) was obtained from the Australian National Algae Culture Collection (ANACC; CSIRO, Hobart, Australia) and grown in f/2 medium (0.2 µm filtered artificial seawater enriched with: 8.82×10^−4^ M NaNO_3_, 3.62×10^−5^ M NaH_2_PO_4_.7H_2_O, trace metal solution and vitamin solution) [36]. Stock cultures were maintained at 20°C under a 12 h:12 h light:dark cycle at 40 µmol photons m^−2^ s^−1^ of photosynthetically active radiation (PAR). Sparging with small bubbles of ambient air ensured that the exchange of atmospheric carbon dioxide and oxygen in the culture medium is maximised. This prevented the steady-state concentrations of these gasses from shifting too far from air equilibrium levels due to photosynthetic and respiratory activities, allowing examination of how irradiance and temperature impact growth. *Nannochloropsis* cells are approximately 3 µm in diameter.

### Photobioreactor Setup

**Figure 1:**
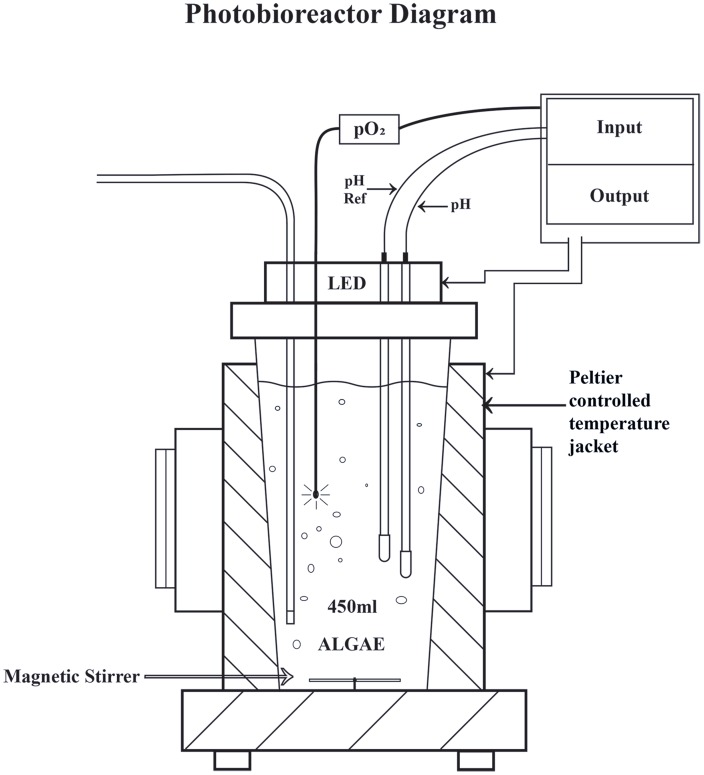
Phenometrics ePBR schematic diagram. Light intensity is controlled using a white light LED array and culture temperature is controlled with a Peltier controlled temperature jacket. The PBR is comprised of a magnetically-stirred chamber, filled with a 10% v/v *N. oculata* stock culture inoculum in f/2 medium. Aeration with ambient air is supplied through a gas dispersion tube. Dissolved oxygen (pO_2_) is measured optically (PyroScience) and pH is measured electrochemically (Phenometrics).

*N. oculata* was cultured in cylindrical photobioreactors (ePBR, Phenometrics, Lansing, MI, USA) with a 450 mL working volume (Figure 1). Experiments were performed over a period of 12 days following an additional 2 day acclimation period of *N. oculata* cultures in the PBRs. Seven PBRs were inoculated with 10% v/v of stock culture. All PBRs were illuminated by LEDs with white light of spectral composition similar to sunlight under a 12 h:12 h light:dark cycle. A comparison between the LED spectral output and the solar spectrum is shown in Figure s1; while the LED provides steady white light across the PAR region, it is particularly rich in blue wavelengths. Vieler *et al*. [37] have recently found evidence for blue light receptors and components of a circadian rhythm in the genome sequences of *N. oceanica* and *N. gaditana*. This makes it likely that *N. oculata* will respond physiologically in some way, when growing under higher intensities of blue light. Whether most or all of that response will be complete within two days, or take much longer, will require further experimental investigation. Sinusoidal irradiance levels during the light phase ranged from 0 to1920 µmol photons m^−2^ s^−1^ PAR, with maximum light intensity occurring at midday. This maximum light intensity corresponds to the approximate solar PAR maximum under direct sunlight in the tropics [38]. Lights were turned on at 06∶00 h and turned off at 18∶00 h. The PBRs were operated in batch mode. Two different temperature regimes were used: constant temperature maintained at 20±1°C and sinusoidal temperature ranging between 15±0.5°C and 25±0.5°C; the peak temperature occurred around dusk (18∶00±30 mins). This 6 h lag between peak ambient light intensity and seawater temperature is common in coastal regions [39]. Four PBRs were operated under constant temperature (*n = 4*) and three PBRs were operated under sinusoidal temperature (*n = 3*). All PBRs were aerated with filtered (oil-free) ambient air that was bubbled into the culture via gas dispersion tubes (product no. 9435–39, porosity D, ACE Glass Inc., Vineland, NJ, USA). However, individual PBRs behave differently due to small variations in sparging rate, mixing and algal culture composition. Measurements of temperature (Phenometrics), pH (Van London pH electrode, Houston, TX, USA) and dissolved oxygen (OXB430-OI bare-fibre oxygen minisensor, Firesting O_2_, PyroScience, Aachen, Germany) were performed continuously in all PBRs. Data were collected every 5 min, so that effects on algal physiology could be closely monitored. Dissolved oxygen measurements were corrected for temperature changes in the PBRs in accordance with theoretical oxygen solubilities in seawater (Figure S2) [40]; pH measurements were additionally corrected for electrode baseline drift.

### Growth Measurements

A direct microscopic cell count was carried out using a haemocytometer (Neubauer, Germany). Cell counts were performed on undiluted 10 µL samples and 5 out of 25 haemocytometer squares were counted twice; each cell count was performed in triplicate on alternate days.

Chlorophyll *a* (Chl *a*) determinations are vital in biofuel research since they provide a fast estimation of algal cell density and physiology [41], [42]. Chl *a* was measured using a modified extraction method [43]. Briefly, a sample of algal culture was filtered (0.2 µm) and the filter extracted in the dark for 24 h in 100% ethanol saturated with MgCO_3_. Samples were then centrifuged and the supernatant analysed spectrophotometrically. Extinction values at 632, 649, 665 and 750 nm were recorded using a UV-Vis spectrophotometer (Varian Cary 50 Bio, Palo Alto, CA, USA). The Chl *a* concentration was calculated using the equation below [43].
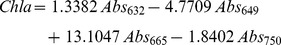



An additional chlorophyll assay was performed, based on Chl *a* fluorescence, with a Turner fluorometer (using the *in vivo* module), where raw fluorescence was measured (Trilogy, Turner Design, Sunnyvale, CA, USA). The excitation wavelength was 485 nm and the fluorescence was detected at >685 nm.

### Fluorescence Measurements

The quantum yield of photosystem II (Y(II)) was determined using a pulse-amplitude modulated fluorometer (Pocket PAM, Walz, Effeltrich, Germany). The Pocket PAM was placed against the outside of the chamber at approximately 1/3 height. Measurements were recorded at six time points throughout the diel cycle: 05∶30 (pre-dawn), 08∶30 (mid-morning), 12∶00 (midday), 16∶15 (mid-afternoon), 17∶30 (pre-dusk) and 18∶15 (early-night). Pocket PAM settings were as follows: blue light, measuring light intensity of ∼0.2 µmol photons m^−2^ s^−1^ PAR, saturation pulse intensity of 2600 µmol photons m^−2^ s^−1^ PAR, and saturation pulse width of 0.8 s.

### Net Photosynthesis Measurements

Net photosynthesis was estimated by measuring the oxygen evolution rates of *N. oculata* at different times of day; analysis was performed at the same time as fluorescence measurements. Aeration of the PBR was temporarily switched off (∼15 mins) whilst minisensors (PyroScience) measured the initial rate of change in dissolved oxygen concentrations. Oxygen evolution was determined by fitting a linear curve to the rate of dissolved oxygen increase. The oxygen evolution rate was normalised against the corresponding Chl *a* content of the *N. oculata* culture.

## Results and Discussion

### *N. oculata* Growth

**Figure 2:**
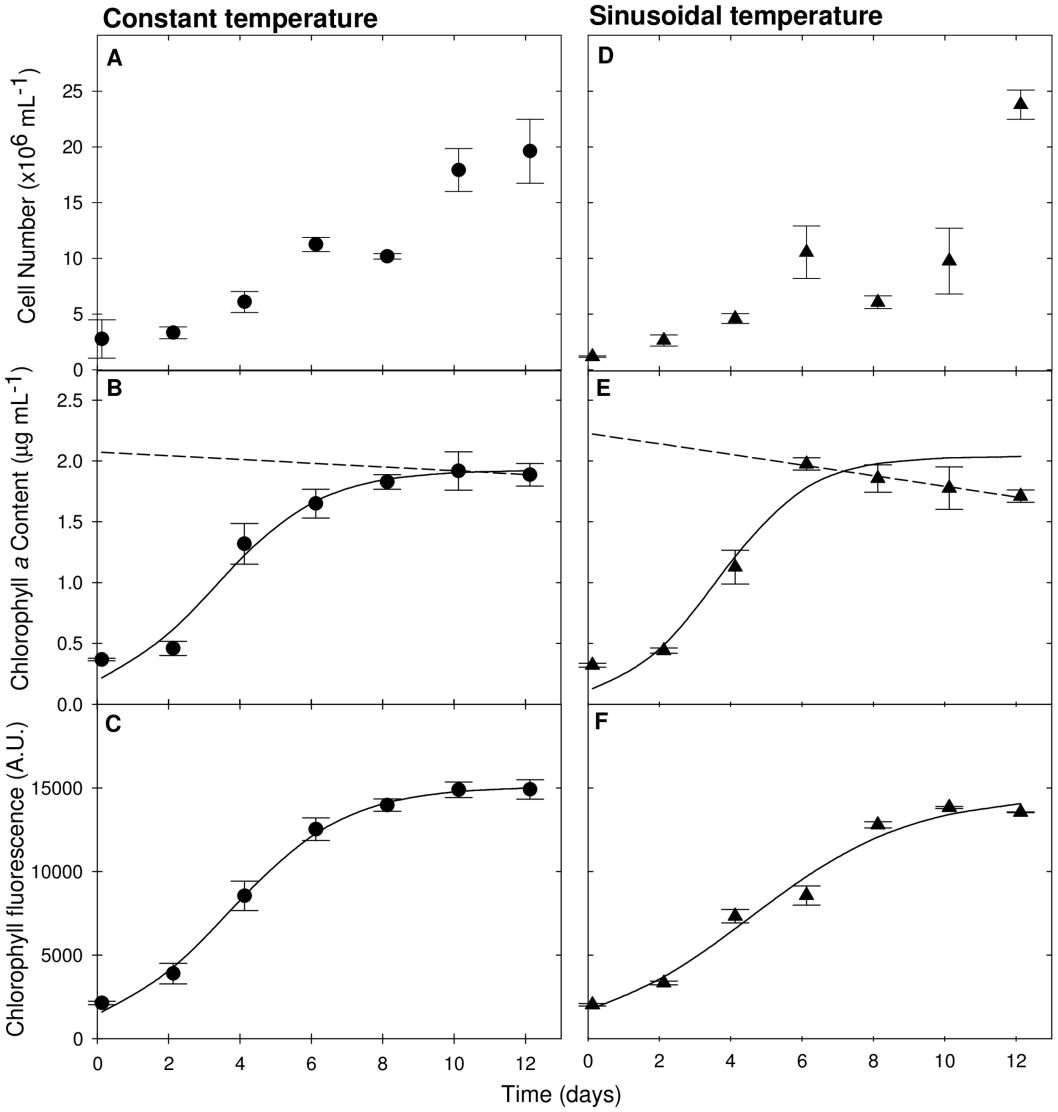
Measurements of *N. oculata* growth. *N. oculata* grown in Phenometrics ePBRs under two different temperature regimes: constant temperature (circles) in A-C and sinusoidal temperature (triangles) in D-F. Growth was measured by microscopic cell counts (A, D), Chl *a* extraction (B, E) and *in vivo* fluorometry (C, F). For constant temperatures n = 4, for sinusoidal temperatures n = 3; error bars represent ±1 standard error. A logistic fit based on the method of least squares [45] was used to model algal growth (B, C, E, F; solid line). Linear regression was used to model subsequent Chl *a* depletion, which occurred after the exponential growth phase (B, C; dotted line).

Three different techniques, microscopic cell counts, *in* *vitro* Chl *a* extraction and *in vivo* Chl *a* fluorometry were used to measure the growth of *N*. *oculata* cultures under constant and sinusoidal temperature regimes (Figure 2). Both temperature treatments showed growth curves with a distinct lag phase (day 0 to day 2), exponential phase (day 2 to day 8) and stationary phase (day 8 to day 12).

Microscopic cell counts proved to be unreliable (Figure 2A and 2D). Cell counting with a haemocytometer is susceptible to various sources of systematic user error; preparation of non-uniform cell suspensions, improper filling of measuring chambers and failure to adopt a convention for counting cells in contact with boundary lines and each other [35]. Notable outliers were obtained on day 8 of the experiment. The percentage error was also large; as high as 15% for constant temperature measurements and 34% for sinusoidal temperature measurements.

*In vitro* Chl *a* extraction measurements (Figure 2B and 2E) are the universally accepted standard for microalgal chlorophyll determination [16]. Chl *a* content is strongly correlated to the density of green microalgal cells [43]. *In vitro* measurements are time-intensive and potentially disruptive to the experiment. *In vivo* Chl *a* fluorescence measurements (Figure 2C and 2F) are a more practical alternative to manual cell counts [44]. The Pearson product-moment correlation coefficient between all growth rate measurements obtained using these two techniques was 0.85. This correlation was higher under constant temperature (Figure 2B and 2C); logistic curve fits [45] give a specific growth rate of 0.60 d^−1^ for Chl *a* extraction, and a comparable 0.62 d^−1^ in the case of *in vivo* Chl *a* fluorescence.

However, this relationship breaks down in the case of sinusoidal temperature (Figure 2E and 2F). Chl *a* extraction measurements show a faster growth rate during the first 6 days of experiment (0.77 d^−1^), followed by a linear depletion of the Chl *a* content. The reliability of this growth rate estimate is questionable, since the logistic curve is based on only five data points (seven in all other cases). The corresponding *in vivo* Chl *a* fluorescence measurements showed no sign of this Chl *a* depletion, and the logistic fit gives a reduced growth rate of 0.44 d^−1^. The most likely reason for this discrepancy is that the *in vivo* Chl *a* fluorescence module cannot distinguish between Chl *a* and Chl *a* breakdown products, such as chlorophyllide *a* [44]. It is therefore generally appropriate to use *in vivo* fluorescence to estimate Chl *a* content during the exponential growth phase, but not during the subsequent stationary phase. Chl *a* extraction will therefore be used as the default measure of *N. oculata* growth in this study.

In general, *N. oculata* growth rates under constant and sinusoidal temperature regimes are very similar. There is some indication that growth may be slightly faster under sinusoidal temperature, but also that this condition may result in faster degradation of Chl *a* during the stationary phase (Figures 2B and 2E). There is minimal difference in cell densities following the exponential growth phase, indicating that the algal culture is either light or nutrient limited. Previous experiments have shown that *Nannochloropsis* can be grown effectively over a wide temperature range: between 15 and 30°C [4], [32], [33]. Our study shows that *N. oculata* also copes well with natural diel variations in temperature between 15 and 25°C. It would appear that fine temperature control is not necessary in industrial *N. oculata* cultivation, apart from the need to control the intense temperature gradients observed in shallow ponds [3]. This result may be different in the case of low carbon dioxide availability or other nutrient limitations. However, it would be premature to draw conclusions at this stage before first examining the effect of diel temperature variations on algal physiology and long-term health.

### Dissolved Oxygen (pO_2_) Profiles

**Figure 3:**
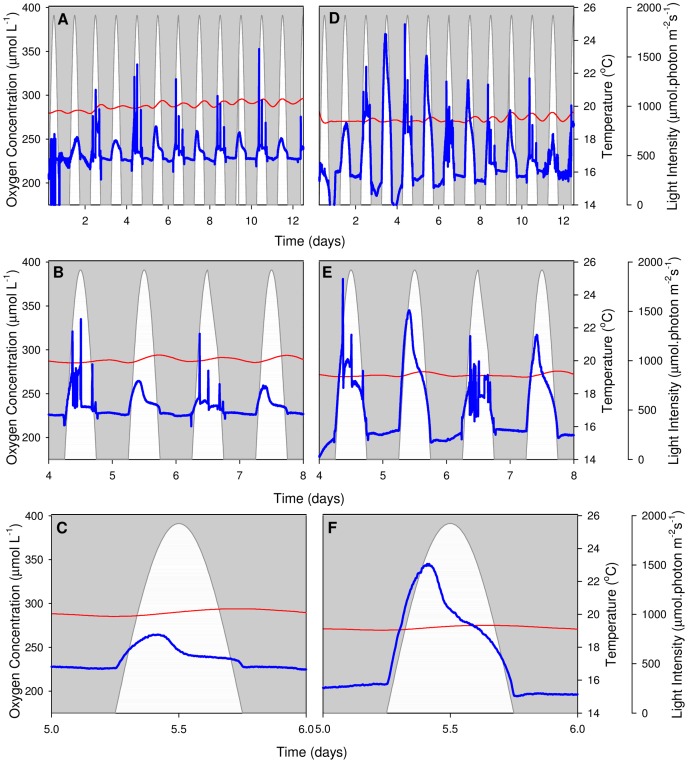
Constant temperature pO_2_ profiles. Dissolved oxygen profiles of *N*. *oculata* when exposed to constant temperature. Two representative PBRs are shown (A, B, C and D, E, F). Top panels (A, D) display the entire experiment, middle panels (B, E) present in more detail the pO_2_ profile during the exponential growth phase (day 4 to day 8), and bottom panels (C, F) highlight a single day (day 5). White regions represent the sinusoidal light regime, thin red lines give the smoothed culture temperature and thick blue lines show the pO_2_ profile. Large spikes on even-numbered days are caused by short interruptions to the air supply during periodic oxygen evolution measurements.

**Figure 4:**
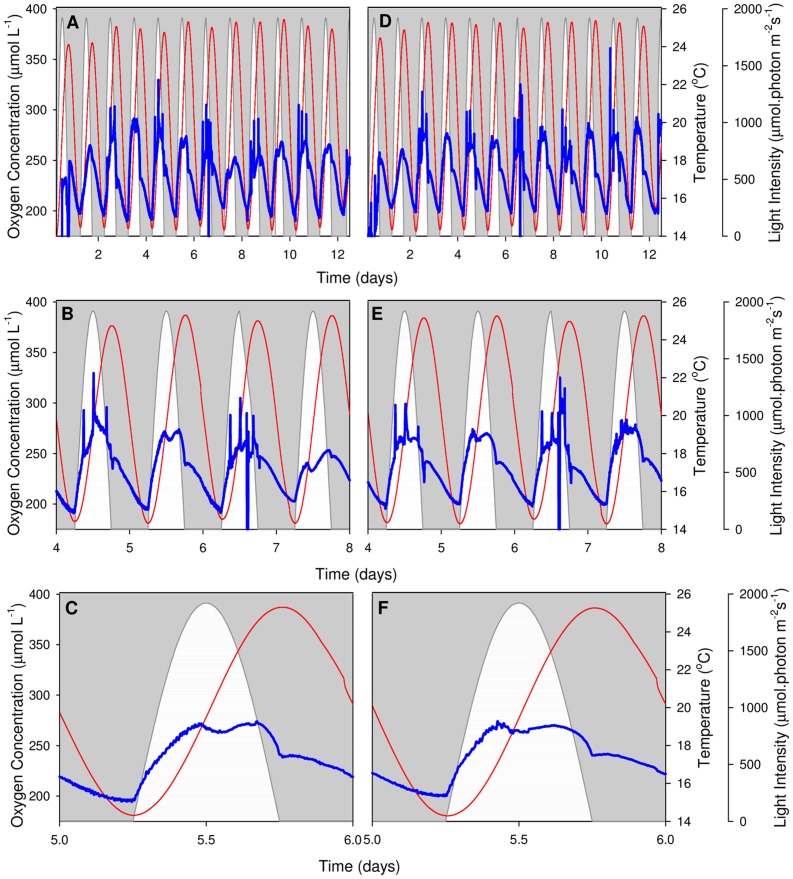
Sinusoidal temperature pO_2_ profiles. Dissolved oxygen profiles of *N*. *oculata* when exposed to sinusoidal temperature. Two representative PBRs are shown (A, B, C and D, E, F). Top panels (A, D) display the entire experiment, middle panels (B, E) present in more detail the pO_2_ profile during the exponential growth phase (day 4 to day 8), and bottom panels (C, F) highlight a single day (day 5). White regions represent the sinusoidal light regime, thin red lines give the smoothed culture temperature and thick blue lines show the pO_2_ profile. Large spikes on even-numbered days are caused by short interruptions to the air supply during periodic oxygen evolution measurements.

*In situ* pO_2_ profiles of *N. oculata* were measured optically under constant temperature (Figure 3) and sinusoidal temperature regimes (Figure 4); all data was post-processed to correct for changes in oxygen solubility at different temperatures. Diel trends are consistent between replicates of identical temperature regimes; however, there is a noticeable difference in the shape of oxygen evolution and cycle duration between the different treatments. The pO_2_ profiles under constant temperature show a 12-hour daylight cycle, compared to the 24-hour cycle evident under the sinusoidal temperature regime.

Under the constant temperature regime, pO_2_ responds strongly to the presence of light, reaching a maximum concentration between 09∶30 and 10∶30 (Figure 3). pO_2_ decreases sharply until the early afternoon (approximately 13∶00), where the rate of decrease slows for a couple of hours, before once again decreasing rapidly until sunset (Figure 3B, D, F). Since the culture is consistently sparged with air there are no obvious signs of oxygen consumption by respiration during the dark period.

Under the sinusoidal temperature regime, pO_2_ values are less dictated by light than those under constant temperature. The increase of pO_2_ at the onset of irradiance at dawn is slower than under constant temperature, despite the fact that the PAR fluctuation stays the same under both treatments. This is because temperature is lower at this early stage of the diel cycle, leading to reduced net photosynthesis [30]. The pO_2_ reaches a primary maximum just before midday (Figure 4B, D, F). It then decreases slightly in accordance with the pattern observed under constant temperature. It is at this stage that a decoupling between pO_2_ and the light intensity occurs. From 14∶00 until the simulated sunset, pO_2_ correlates strongly with the sinusoidal temperature cycle, reaching a secondary maximum shortly prior to the temperature maximum at 18∶00. This maximum can be explained by the increased net photosynthesis at higher temperatures [30]. It is nevertheless surprising that pO_2_ increases while PAR is decreasing, since PAR provides the energy for the water-splitting reaction. Following the secondary maximum, pO_2_ levels decrease rapidly until night, but there is not enough time for them to return to the baseline values of dawn that day. During the subsequent dark period, the rate of pO_2_ decrease slows and pO_2_ levels eventually return to initial (pre-sunrise) levels due to the combined influence of respiration and air sparging. More detailed studies are needed to determine if there is evidence for an initial rapid O_2_ uptake after cessation of illumination followed by a lower rate of respiration [46].

Absolute pO_2_ values are difficult to interpret because pO_2_ is a function of physical (sparging) and biological (photosynthesis and respiration) processes. The physical sparging process is constant throughout the day and generally acts to remove excess oxygen produced by net photosynthesis. Dissolved oxygen goes down during the second half of the light period because the rate of oxygen removal by sparging is faster than the rate of oxygen production by net photosynthesis. This effect is exemplified by the significant variations in pO_2_ amplitudes between Figures 3B and 3D. Although all PBRs use identical air dispersion tubes and air pressures, variations are introduced due to the propensity of *N. oculata* to colonise small apertures such as those in glass sinters (sparging bars), leading to time and PBR dependent changes in the air dispersion rate; this limitation will be addressed in future experiments. It is, however, possible to compare trends in pO_2_ amplitude in selected PBRs (Figure S3: constant temperature regime; Figure S4: sinusoidal temperature regime). Under both temperature regimes, pO_2_ concentration is highest during the exponential growth phase (day 3 and day 7). This effect will be explored in more detail using net photosynthesis measurements. There is also a temporal shift in the pO_2_ maxima under constant temperature; in general, the maximum occurs earlier, and it is more pronounced, when the algal growth rate is faster (Figure S3). This result indicates that *N. oculata* cells in the exponential growth phase require less light to produce the same amount of oxygen by photosynthesis. The corresponding sinusoidal temperature results are influenced by both light intensity and temperature variations (Figure S4). The hysteresis effect is clear in Figure S3, where the degree of photosynthetic down-regulation is greater in the afternoon than in the morning. This is caused when oxygen evolution is measured under increasing steps in irradiance, rather than decreasing steps in irradiance, as occurs in the afternoon. The shape of the hysteresis is dependent upon irradiance; however as temperature alters the rate of D1 protein repair [47], this could also alter the hysteresis.

### pH Profiles

**Figure 5:**
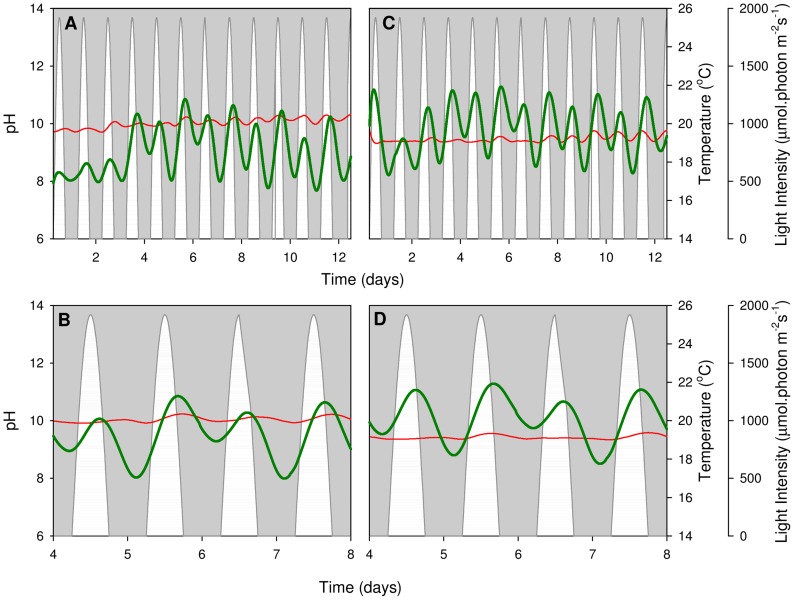
Constant temperature pH profiles. Smoothed (moving average) pH profiles of *N*. *oculata* when exposed to constant temperature. Two representative PBRs are shown (A, B and C, D). Top panels (A, C) display the entire experiment, while bottom panels (B, D) present in more detail the pH profile during the exponential growth phase (day 4 to day 8). White regions represent the sinusoidal light regime, thin red lines give the smoothed culture temperature and thick green lines show the pH profile.

**Figure 6:**
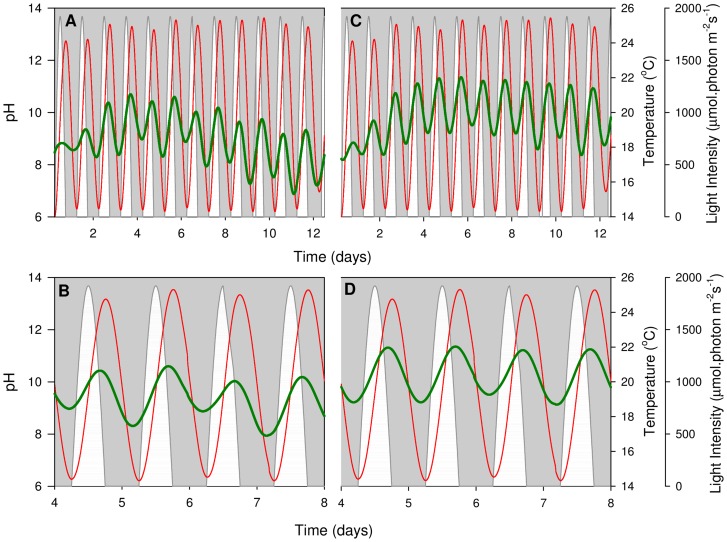
Sinusoidal temperature pH profiles. Smoothed (moving average) pH profiles of *N*. *oculata* when exposed to sinusoidal temperature. Two representative PBRs are shown (A, B and C, D). Top panels (A, C) display the entire experiment, while bottom panels (B, D) present in more detail the pH profile during the exponential growth phase (day 4 to day 8). White regions represent the sinusoidal light regime, thin red lines give the smoothed culture temperature and thick green lines show the pH profile.

pH profiles were determined electrochemically under constant temperature (Figure 5) and sinusoidal temperature regimes (Figure 6); all data were smoothed and post-processed to account for probe drift and temperature variations. Under both temperature regimes, pH fluctuations occur in 24-hour cycles, with higher pH levels measured during the light phase. At night, a decrease in pH is seen due to a decrease in the pO_2_:pCO_2_ ratio as a result of the absence of photosynthesis and presence of respiration [48]. The maximum rate of pH increase occurs at approximately 10∶00 under constant temperature and just before midday under sinusoidal temperature. This strong correlation between pH increase and maximum pO_2_ concentration (c.f. Figures 3, 4, 5 and 6) indicates that both parameters provide a reasonable estimate for the diel variations in photosynthesis rate.

Further to this, a 48 h pattern occurs where the pH shows a more gradual nocturnal decrease on every even-numbered day. This trend is more apparent under the constant temperature regime (Figure 5B, D), where diel temperature variations do not affect the pH measurements. The 48 h trend coincides with the oxygen evolution measurements that took place on alternate days and caused interruptions to the air dispersion.

*N. oculata* can tolerate alkaline pH fluctuations, since growth occurs between pH 8 and pH 13 [4], [35]. The experimental variations in this study are comfortably within this pH tolerance range, indicating that pH variations did not limit *N. oculata* growth.

Figures 5A, C and 6A, C show curved responses to time in the overall pH amplitudes of cultures; this pattern is more prevalent in the sinusoidal regime. This result was also observed in the *in vitro* Chl *a* measurements (Figure 2B, E): the curvature of the pH response to time appears to follow the Chl *a* density. A strong pH (and Chl *a* density) decrease was observed in the last four days of the experiment under sinusoidal temperature conditions, whereas the constant temperature curve appears more linear. As discussed previously (Results and Discussion; *N. oculata* growth), the sinusoidal temperature regime likely leads to faster rates of Chl *a* breakdown in the stationary growth phase (Figure 2E). This increases the respiration-to-photosynthesis ratio, leading to the observed reductions in pH levels (Figure 6A, C) [35]. The pH data show no evidence of an increase in culture medium pH (at a given CO_2_ partial pressure) with algal growth, which could result from the production of alkali in the assimilation of external nitrate into organic nitrogen [49], [50].

### Quantum Yield of Photosystem II and Net Photosynthesis

**Figure 7:**
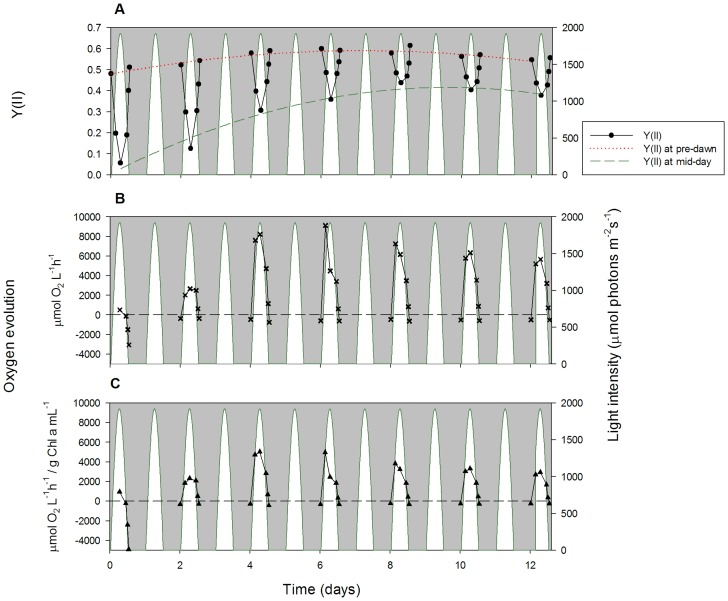
Quantum yield of photosystem II and net photosynthesis under constant temperature. Algal physiology parameters, including: the quantum yield of photosystem II (YII) measured using PAM fluorometry (A, circles), the rate of oxygen concentration change when positive, representing net photosynthesis (B, crosses), and net photosynthesis normalised against Chl *a* content (C, triangles). Data were collected on alternate days, at 6 time points throughout the diel cycle. White areas represent the sinusoidal light regime.

**Figure 8:**
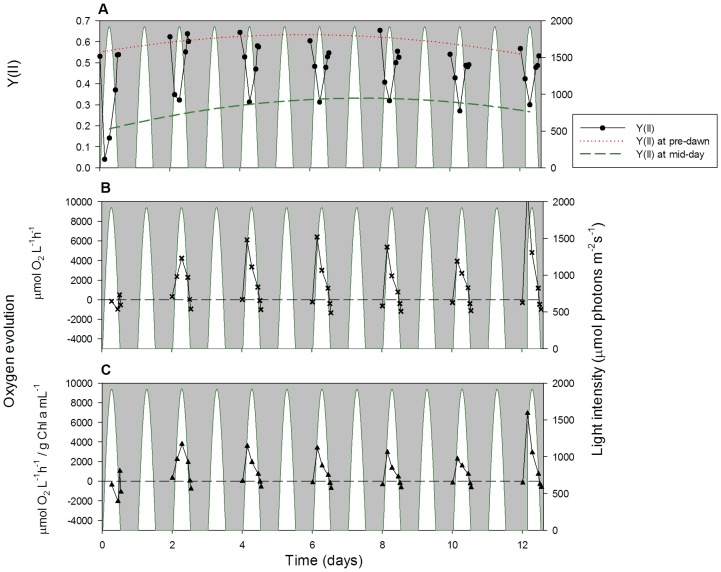
Quantum yield of photosystem II and net photosynthesis under sinusoidal temperature. Algal physiology parameters, including: the quantum yield of photosystem II (YII) measured using PAM fluorometry (A, circles), the rate of oxygen concentration change when positive, representing net photosynthesis (B, crosses), and net photosynthesis normalised against Chl *a* content (C, triangles). Data were collected on alternate days, at 6 time points throughout the diel cycle. White areas represent the sinusoidal light regime.

Algal photosynthetic performance was determined using Pocket PAM fluorescence and net photosynthesis measurements (Figure 7 for constant temperature and Figure 8 for sinusoidal temperature). These measurements were very consistent between different PBRs (see Figure S5 and Figure S6 for additional replicates).

The PAM fluorescence parameter chosen was Y(II), the light-adapted effective quantum yield of photosystem II (PSII), i.e. the efficiency at which light absorbed by PSII is used for reduction of the primary quinone electron acceptor, Q_A_ [51]. Sforza *et al*. [23] found overnight estimates of maximum quantum yield of PSII to average 0.6 in *Nannochloropsis salina*, which is similar to our results obtained in *N. oculata*. The V-shaped pattern of Y(II) in Figures 7A and 8A represents the decreasing effective quantum yield of PSII as light intensities increase during the day, followed by increased effective quantum yield at lower light intensities in the evening. This hysteresis is linked with the widely observed afternoon depression of Y(II) [34]. It has been shown that *Nannochloropsis* sp. responds to variable light intensity by regulating its pigment composition and the ratio between enzyme concentration and irradiation, in an attempt to minimise photodamage [48], [52], [53]. Figures 7 and 8 illustrate the shade-adapted response that needs to be identified when Y(II) is measured in PBR vessels with a heterogeneous light climate (also shown in Figure S5 and Figure S6). The lowest effective quantum yield is represented by the Y(II) minima at midday.

The pre-dawn Y(II) measurements are similar between the constant and sinusoidal temperature regimes, although the sinusoidal temperature quantum yields are slightly higher, with values ranging from 0.52 to 0.65 (Figure 8A), compared with 0.49 to 0.60 under constant temperature (Figure 7A). In general, pre-dawn Y(II) values increase slowly over the first eight days of experiment before stabilising. Under constant temperature, pre-dawn Y(II) is 0.49±0.01 (n = 4) on day 0 and 0.58±0.01 (n = 4) on day 8; under sinusoidal temperature, the corresponding Y(II) increase is between 0.49±0.04 (n = 3) on day 0 and 0.63±0.02 (n = 3) on day 8. Mid-day Y(II) values also increase over time with increasing algal cell density and remain very similar between the two temperature regimes. Under constant temperature, mid-day Y(II) is a very low 0.06±0.02 on day 0, but increases to 0.34±0.06 on day 8; similarly, mid-day Y(II) under sinusoidal temperature starts at 0.08±0.06 on day 0 and recovers to 0.33±0.06 by day 8.

It is highly likely that the *N. oculata* photosystem II in the early afternoon under the sinusoidal temperature regime becomes photodamaged by both the exceedingly high light intensity and the elevated temperature. This view is supported by the apparent hysteresis observed in the Y(II) V-shaped curves; excess temperature after midday has a negative influence on Y(II), relative to the lower temperatures in the morning. Nevertheless, sinusoidal Y(II) values consistently recover by pre-dawn, indicating that sinusoidal temperature (Figure 8A) may stimulate faster photosystem recovery in low light and darkness compared with constant temperature (Figure 7A). During the dark phase, the sinusoidal temperature regime begins with a relatively high temperature and finishes at the temperature minimum of 15°C by pre-dawn. Future experiments will investigate whether these temperature changes really do assist photosystem recovery.

The light climate within the PBR is heterogeneous; both with time as the culture ages and vertically with increased absorption in the upper regions of the PBR and more shading towards the bottom of the chamber. The incremental increase of Y(II) values at midday is potentially caused by greater levels of self-shading due to the increased cell density (through increased cell count numbers and Chl *a* content; Figure 2) during *N. oculata* growth. As cultures increase in density, mixing becomes more important whereby cells are moved between areas of high light to more shaded regions of the PBR; this can provide an overall increase in growth. Net photosynthesis rates, based on oxygen evolution measurements (Figures 7B and 8B), provide an accurate representation of the growth rate [35], particularly when normalised against the corresponding Chl *a* content of the cells (Figures 7C and 8C) in agreement with Flameling & Kromkamp [54]. Under constant temperature, maximum net photosynthesis typically occurs during the highest light intensity at midday; the exceptions are days 6 and 8, where the maxima are at mid-morning. Under sinusoidal temperature, maximum net photosynthesis occurs consistently at mid-morning from day 4 of the experiment. This is the result of the reversible down-regulation of photosynthesis by photoinhibition, which occurs after midday and is caused by the combination of high light intensity and temperature. From day 8, the net photosynthesis and Y(II) no longer recover to their initial mid-morning peaks, suggesting that photodamage has occurred due to the combined long-term light and temperature stress (Figure 7A and 8A). Afternoon net photosynthesis values also show an additional hysteresis under the sinusoidal temperature regime, with the oxygen evolution rate remaining high later on in the day under the influence of higher temperatures (Figure 8B). The respiration that occurs after sunset is greater than during pre-dawn under the sinusoidal temperature regime, but not at constant temperature (Figure 7B). This is likely to reflect the relationship between mitochondrial respiration and temperature given the much higher temperature maintained at the end of the light period in the sinusoidal regime [46].

The trends in maximum normalised net photosynthesis (Figure 7C and 8C), pre-dawn Y(II) (Figure 7A and 8A) and algal growth rates measured as a function of *in vitro* Chl *a* content (Figure 2B and 2E) are all consistent across both temperature regimes. All of these measurements provide a reasonable estimate of *N. oculata* growth rates, but they also combine to deliver a more detailed understanding of the physiology of algal cells exposed to different environmental conditions. For example, it becomes clear that the reduction in Chl *a* content under sinusoidal temperature stems from the combined photoinhibitive effects of high light intensity and temperature in the early-to-mid afternoon. Similarly, high afternoon and evening temperatures in the sinusoidal regime lead to a positive hysteresis in the oxygen evolution rate, resulting in higher relative photosynthetic activity towards the end of the day, which may increase the possibility of full pre-dawn Y(II) recovery.

## Conclusions

The growth and physiology of the biofuel candidate microalgal species *N. oculata* was examined under two different temperature regimes: constant temperature at 20°C and sinusoidal temperature varying between 15°C and 25°C. The experiments were performed in a matrix of seven PBRs (ePBR, Phenometrics, Lansing, MI, USA; Figure 1), under sinusoidal light intensity varying between 0 and 1920 µmol photons m^−2^ s^−1^ PAR.

The *N. oculata* growth rates, measured as a function of Chl *a* accumulation, were similar under the two regimes (Figure 2). This result implies that no temperature control is necessary to compensate for ±5°C sinusoidal temperature variations in industrial *N. oculata* growth facilities. However, a decline in Chl *a* content under the sinusoidal temperature regime (Figure 2E) raised questions about the long-term health and physiology of *N. oculata* cultures. Dissolved oxygen (Figures 3 and 4) and pH (Figures 5 and 6) profiles showed that algal cells were more active beyond midday under elevated afternoon and early-evening temperatures of the sinusoidal temperature regime.

Furthermore, measurements of the midday Y(II) and the corresponding net photosynthesis rates (Figures 7 and 8) showed that photosynthesis in *N. oculata* cells is down-regulated to a greater extent under the combined influence of high light intensity and temperature in the early afternoon of the sinusoidal temperature regimes. However, the sinusoidal temperature regime also facilitated enhanced photosystem recovery at night, resulting in slightly higher pre-dawn Y(II) values. This effect is likely to be related to increased photosynthetic activity after midday under sinusoidal temperature. The combined effects of increased photosynthetic down-regulation during the day and improved Y(II) recovery under low light and at night appear to cancel each other out, resulting in similar growth rates under sinusoidal and constant temperature regimes. Improved understanding of the photobiological mechanisms that govern these processes is required. This study demonstrates that our PBRs, with their environmental control features and high-resolution monitoring of algal growth and physiology, have the potential to answer many of the unresolved questions in algal biofuel production.

## Supporting Information

Figure S1 **Comparison of spectral irradiance by Phenometrics ePBR LED (red) with solar spectrum (blue).** Total PAR was normalised to 500 µmol photons m^−2^ s^−1^; the ePBR LED provides a steady white output across the PAR range, but it is particularly rich in blue wavelengths, with a strong peak at 450 nm.
(TIF)

Figure S2 **Effect of temperature correction on baseline pO_2_ measurements.** Raw experimental baseline measurements (black lines), taken in the f/2 medium in the absence of *N. oculata*, were corrected for sinusoidal variations in oxygen solubility at different temperatures to give the temperature-corrected baseline measurement (blue lines); two representative PBRs shown.
(TIF)

Figure S3 **pO_2_ profile evolution at constant temperature.** Data were recorded on alternate days between 05∶00 and 24∶00 for one representative PBR.
(TIF)

Figure S4 **pO_2_ profile evolution at sinusoidal temperature.** Data were recorded on alternate days between 05∶00 and 24∶00 for one representative PBR. Strong hysteresis in pO_2_ during second part of the day results from increasing temperature.
(TIF)

Figure S5 **Quantum yield of photosystem II and net photosynthesis under constant temperature (second replicate).** Algal physiology parameters, including: the quantum yield of photosystem II (YII) measured using PAM fluorometry (A, circles), the rate of oxygen concentration change when positive, representing net photosynthesis (B, crosses), and net photosynthesis normalised against Chl *a* content (C, triangles). Data were collected on alternate days, at 6 time points throughout the diel cycle. White areas represent the sinusoidal light regime.
(TIF)

Figure S6 **Quantum yield of photosystem II and net photosynthesis under sinusoidal temperature (second replicate):** Algal physiology parameters, including: the quantum yield of photosystem II (YII) measured using PAM fluorometry (A, circles), the rate of oxygen concentration change when positive, representing net photosynthesis (B, crosses), and net photosynthesis normalised against Chl *a* content (C, triangles). Data were collected on alternate days, at 6 time points throughout the diel cycle. White areas represent the sinusoidal light regime.
(TIF)
