# Supplementary figures and images for: The Effect of Diel Temperature and Light Cycles on the Growth of Nannochloropsis oculata in a Photobioreactor Matrix
Source: PLoS One. 2014 Jan 20;9(1):e86047. doi: 10.1371/journal.pone.0086047 (PMC3896454; doi:10.1371/journal.pone.0086047)

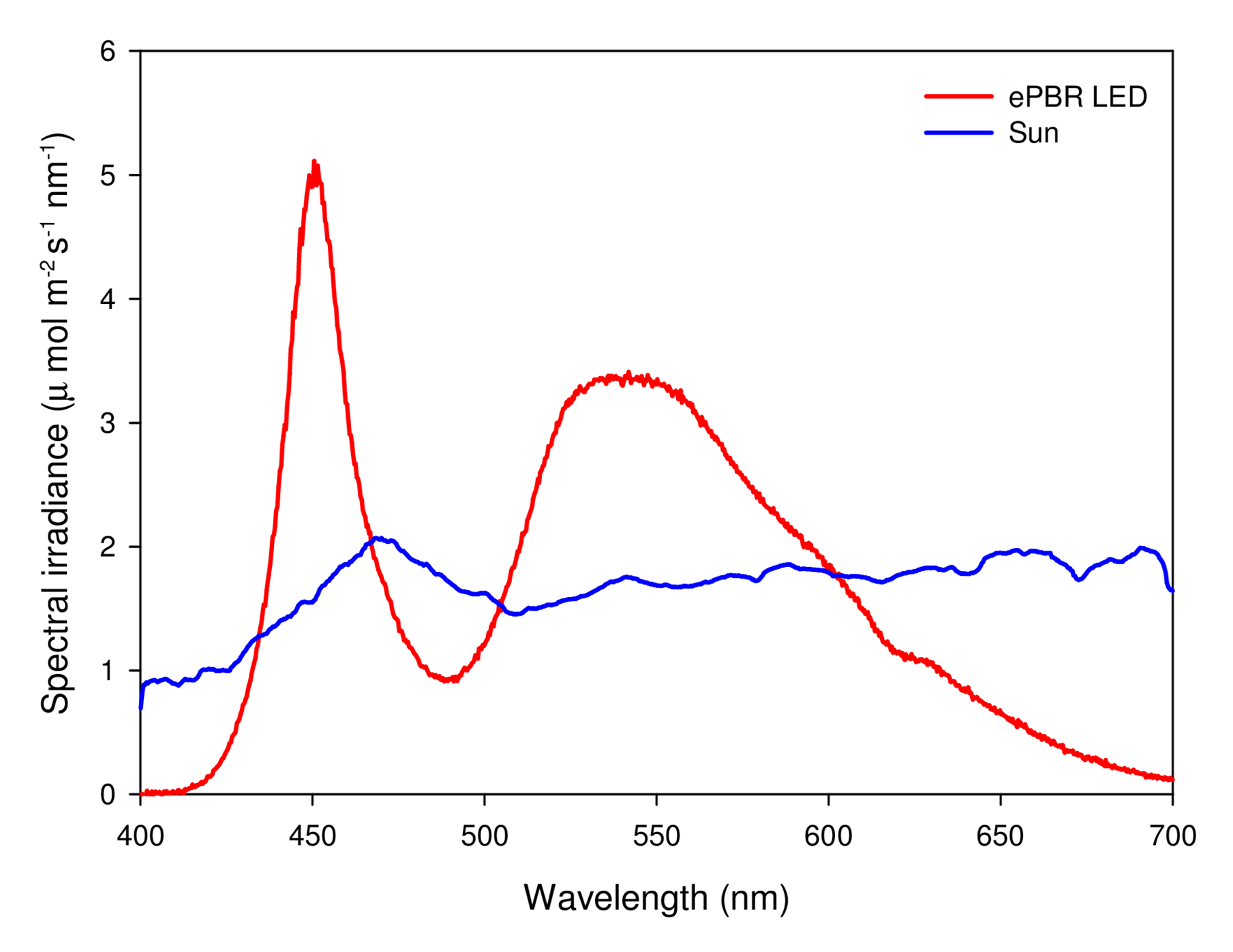

Supplement: Figure S1 — Comparison of spectral irradiance by Phenometrics ePBR LED (red) with solar spectrum (blue). Total PAR was normalised to 500 µmol photons m−2 s−1; the ePBR LED provides a steady white output across the PAR range, but it is particularly rich in blue wavelengths, with a strong peak at 450 nm. (TIF) [file pone.0086047.s001.tif]

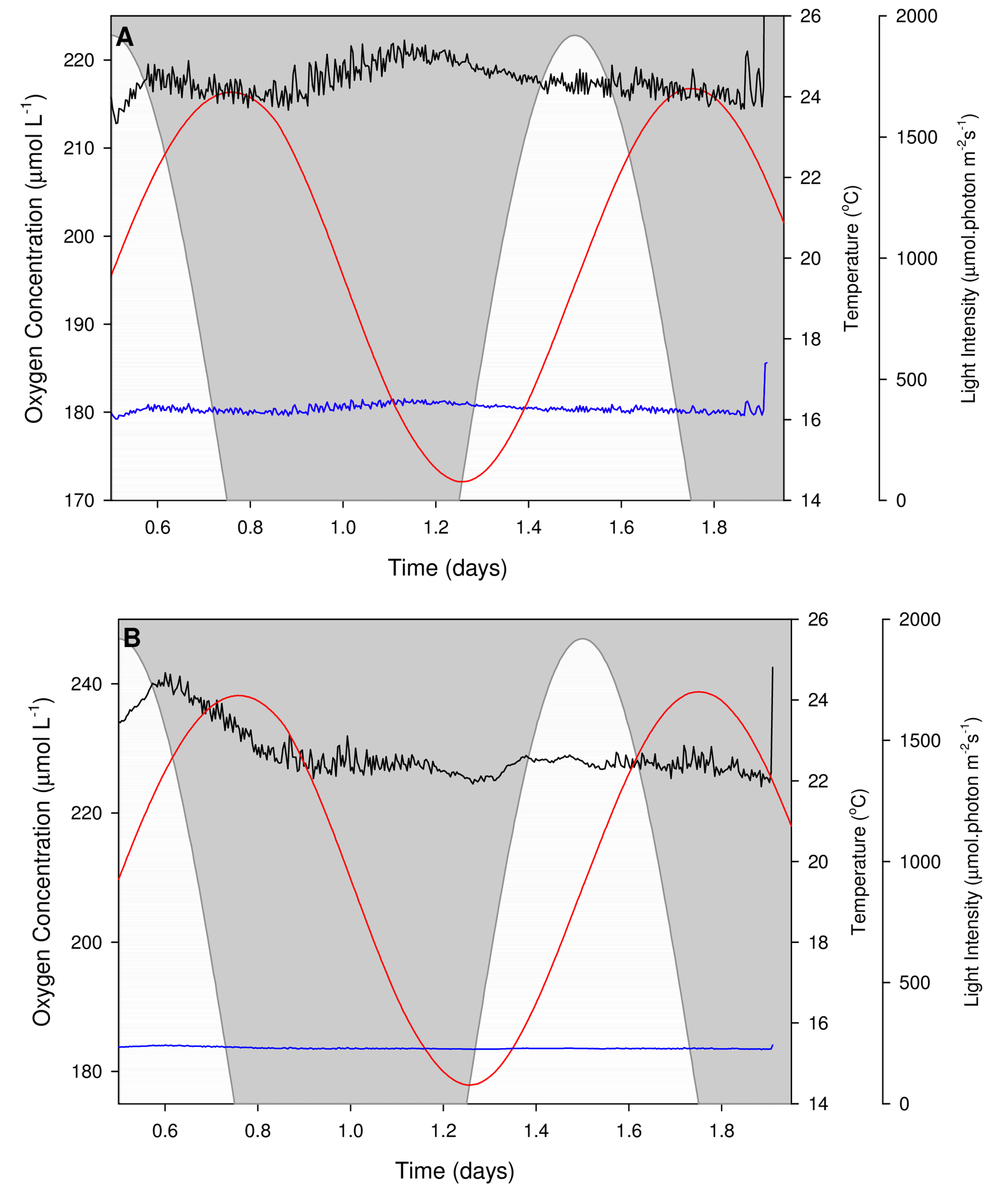

Supplement: Figure S2 — Effect of temperature correction on baseline pO2 measurements. Raw experimental baseline measurements (black lines), taken in the f/2 medium in the absence of N. oculata, were corrected for sinusoidal variations in oxygen solubility at different temperatures to give the temperature-corrected baseline measurement (blue lines); two representative PBRs shown. (TIF) [file pone.0086047.s002.tif]

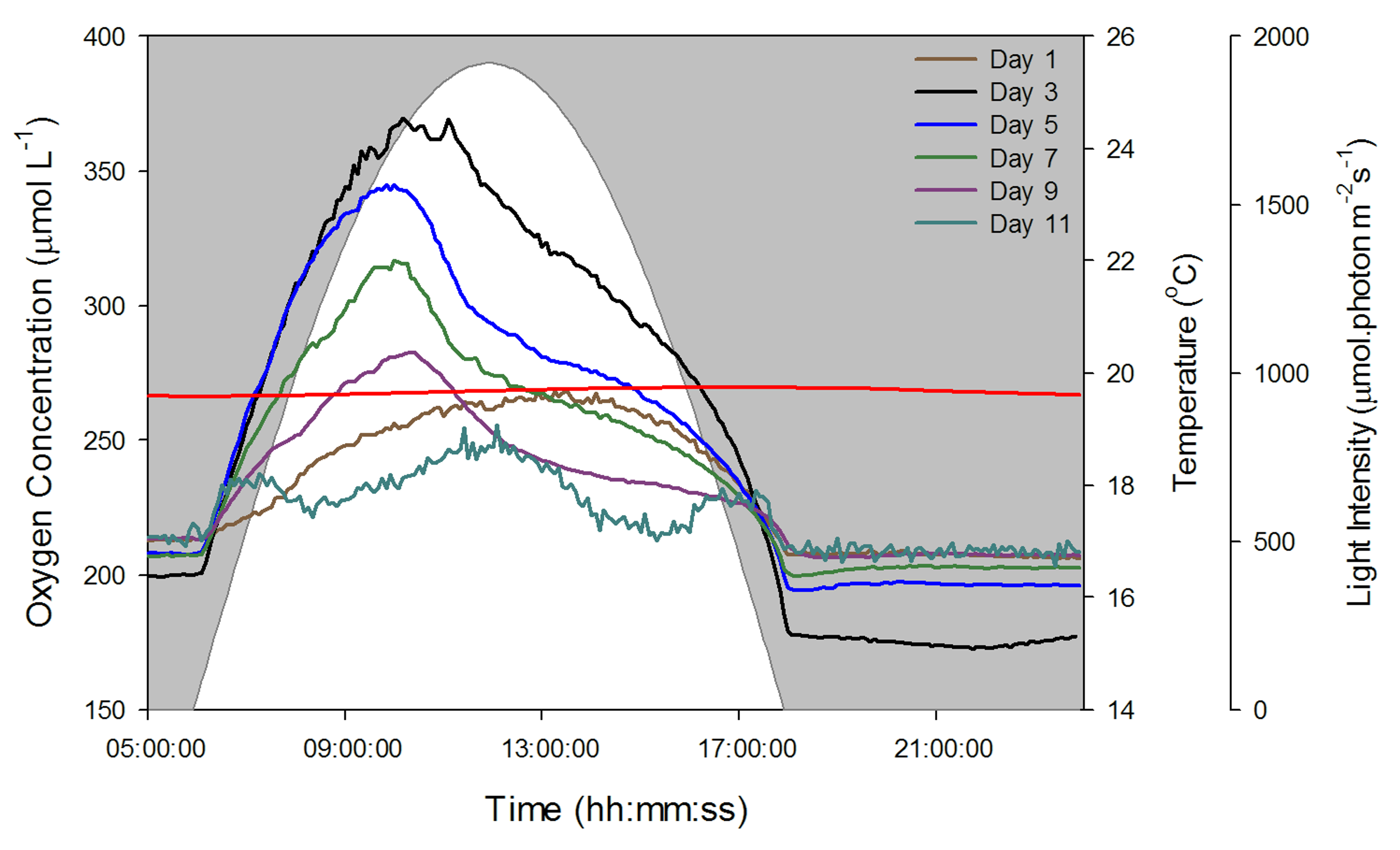

Supplement: Figure S3 — pO2 profile evolution at constant temperature. Data were recorded on alternate days between 05∶00 and 24∶00 for one representative PBR. (TIF) [file pone.0086047.s003.tif]

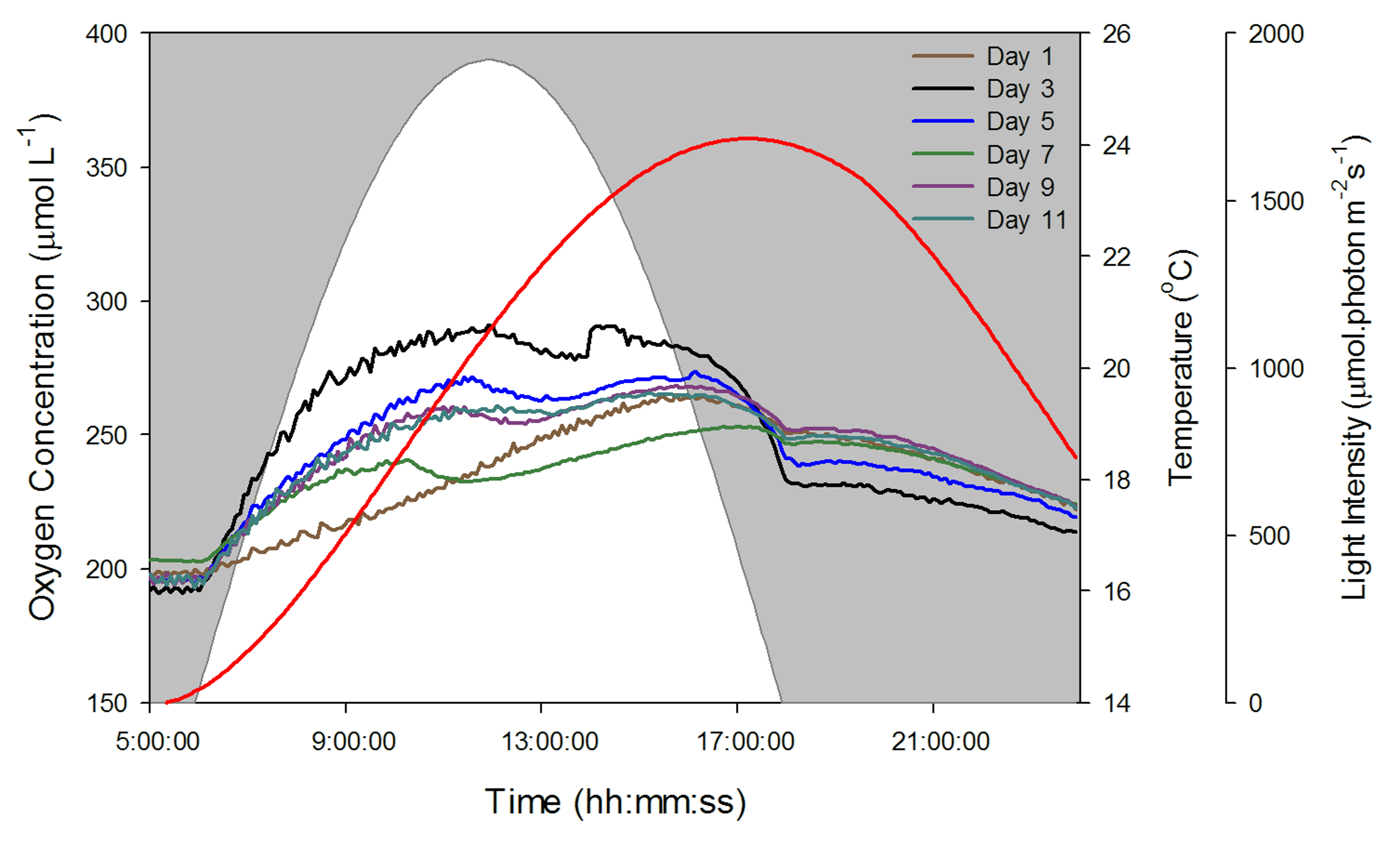

Supplement: Figure S4 — pO2 profile evolution at sinusoidal temperature. Data were recorded on alternate days between 05∶00 and 24∶00 for one representative PBR. Strong hysteresis in pO2 during second part of the day results from increasing temperature. (TIF) [file pone.0086047.s004.tif]

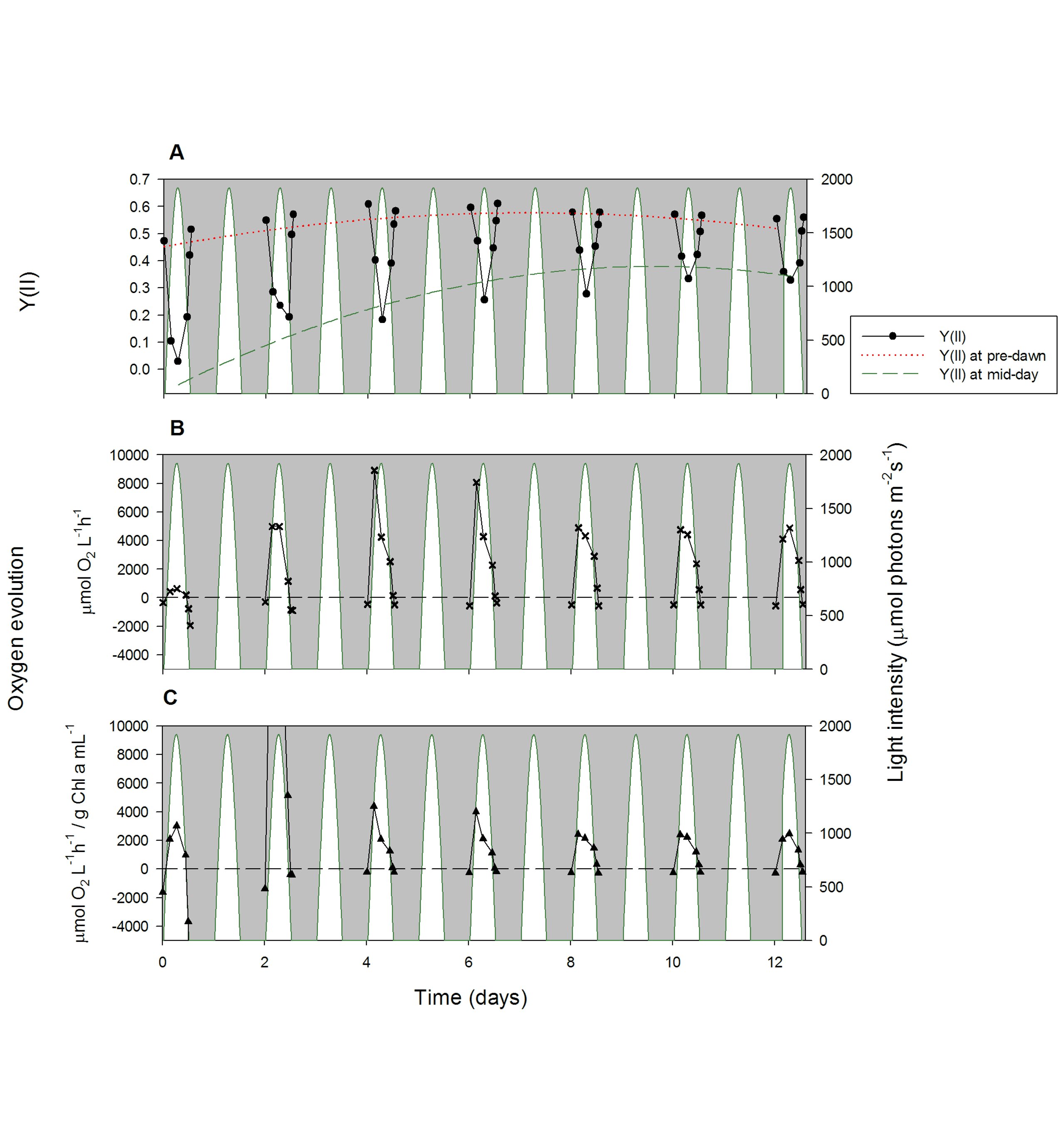

Supplement: Figure S5 — Quantum yield of photosystem II and net photosynthesis under constant temperature (second replicate). Algal physiology parameters, including: the quantum yield of photosystem II (YII) measured using PAM fluorometry (A, circles), the rate of oxygen concentration change when positive, representing net photosynthesis (B, crosses), and net photosynthesis normalised against Chl a content (C, triangles). Data were collected on alternate days, at 6 time points throughout the diel cycle. White areas represent the sinusoidal light regime. (TIF) [file pone.0086047.s005.tif]

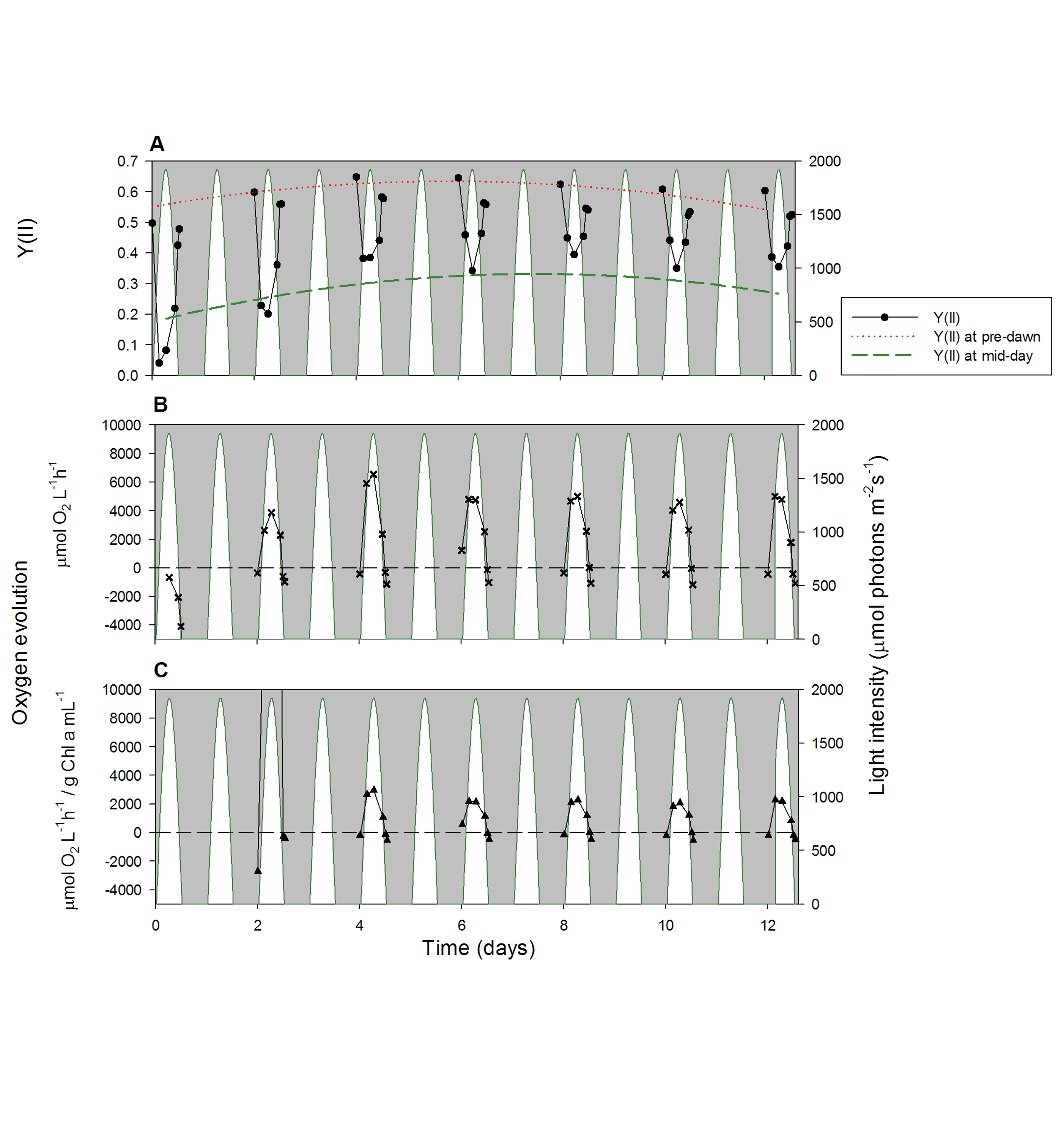

Supplement: Figure S6 — Quantum yield of photosystem II and net photosynthesis under sinusoidal temperature (second replicate): Algal physiology parameters, including: the quantum yield of photosystem II (YII) measured using PAM fluorometry (A, circles), the rate of oxygen concentration change when positive, representing net photosynthesis (B, crosses), and net photosynthesis normalised against Chl a content (C, triangles). Data were collected on alternate days, at 6 time points throughout the diel cycle. White areas represent the sinusoidal light regime. (TIF) [file pone.0086047.s006.tif]
